# Successful treatment of an NDM-5-producing, carbapenem-resistant *Salmonella* Kentucky ST198 infection with aztreonam and ceftazidime-avibactam in a patient with acute erythroid leukemia: a case report

**DOI:** 10.1186/s12879-026-13519-9

**Published:** 2026-06-10

**Authors:** Yingcheng Qin, Li Lin, Hengrui Zhao, Xiumei Hu

**Affiliations:** 1 Department of Laboratory Medicine, Guangdong Provincial Key Laboratory of Precision Medical Diagnostics, Guangdong Engineering and Technology Research Center for Rapid Diagnostic Biosensors, Guangdong Provincial Key Laboratory of Single-Cell and Extracellular Vesicles, Nanfang Hospital, Southern Medical University, Guangzhou, 510515 P. R. China; 2 Guangdong Provincial Clinical Research Center for Laboratory Medicine, Guangzhou, P. R. China

**Keywords:** Carbapenem-resistant, *S.* Kentucky, AML-M6, NDM-5, Drug combination

## Abstract

**Introduction:**

*Salmonella enterica* serotype Kentucky (*S*. Kentucky) is a globally distributed food-borne zoonotic pathogen. It is increasingly associated with multi-drug resistance (MDR), notably to ciprofloxacin, a trend largely attributed to antibiotic overuse in humans and livestock.

**Case presentation:**

An acute erythroid leukemia (AML-M6) man was found to be infected with carbapenem-resistant (CR) *Salmonella enterica* after chemotherapy. *Salmonella* serum agglutination test showed that the strain belonged to *S*. Kentucky. Antimicrobial susceptibility test showed that this strain is an MDR strain (resistant to carbapenems, ciprofloxacin, third-generation cephalosporins). Carbapenemase production was assessed via a colloidal gold immunochromatography assay and indicated expression of an NDM enzyme. Whole genome sequencing (WGS) revealed that this strain carried blaNDM-5, blaCTXM-55, and blaTEM-1 genes. Based on the identification of an NDM carbapenemase, in vitro synergy testing was performed, which demonstrated a synergistic effect between aztreonam and ceftazidime-avibactam. The patient was successfully treated with this combination regimen and recovered.

**Conclusions:**

This report describes an unusual infection caused by an NDM-5-producing *S*. Kentucky in an immunocompromised host. It underscores the necessity of employing combined phenotypic, immunologic, and molecular diagnostics to rapidly elucidate resistance mechanisms in such complex cases. Guided by this comprehensive analysis, the combination of aztreonam and ceftazidime-avibactam proved to be an effective, mechanism-based therapeutic strategy, highlighting its value in managing challenging infections in patients with hematological malignancies.

**Clinical trial number:**

Not applicable.

**Supplementary Information:**

The online version contains supplementary material available at 10.1186/s12879-026-13519-9.

## Introduction

*S*. Kentucky is a non-typhoidal *Salmonella* (NTS) serovar with global distribution, predominantly isolated from poultry and livestock [1]. However, it has emerged as a significant human pathogen in recent years [2]. Of particular concern is the global dissemination of the MDR clone ST198, which poses a substantial threat to public health [3]. In immunocompromised hosts, particularly patients with hematologic malignancies or chemotherapy-induced neutropenia, *Salmonella* can lead to severe, life-threatening infections such as gastroenteritis and bacteremia, which are often complicated by secondary foci.

Infections with MDR *Salmonella* leave patients with limited treatment options and they place a significant burden on healthcare systems. This threat is magnified in patients with acute myeloid leukemia (AML), whose prolonged chemotherapy-induced neutropenia renders them highly susceptible to invasive bacterial infections [4]. A global study of drug-resistant *Salmonella* from 1900 to 2023 shows that it was prevalent across six continents (North America, South America, Oceania, Asia, Europe, Africa). Among the 208,233 *Salmonella* genomes analyzed in this study, China emerged a hotspot for drug resistance, with the resistance rates to fluoroquinolones reaching as high as 83.39%, β-lactam resistance reaching 57.30%, and MDR 69.37%. Among the 20 dominant serovars, the 12,803 *S.* Kentucky genomes exhibited a fluoroquinolone non-susceptibility rate of 99.98% and a β-lactam resistance rate of 13.91% [5]. Likewise, a retrospective study analyzing 10,136 *Salmonella* isolates from 23 Chinese provinces (1994–2021) identified 223 (2.2%) as *S.* Kentucky, ranking it the ninth most common serovar. Most of these *S*. Kentucky isolates originated from chickens (53.76%) and humans (38.71%), and exhibited striking resistance rates: 89.69% to fluoroquinolones, 47.53% to cephalosporins, and 92.38% to multiple antimicrobial classes [6]. Notably, carbapenem resistance among these isolates was exceedingly rare, with a rate of only 0.45%. This is further underscored by WGS data from 9,557 *S.* Kentucky strains, which identified 35,594 antimicrobial resistance genes associated with 12 different classes of antibiotics. Within this vast repertoire, carbapenemase-encoding genes accounted for merely 16 [6].

Herein, we describe an unusual case of CR *S*. Kentucky gastroenteritis in a neutropenic patient with AML-M6. The strain harbored blaNDM-5, blaCTXM-55, and blaTEM-1, as confirmed by WGS. In vitro synergy testing demonstrated efficacy of the aztreonam-ceftazidime-avibactam combination, which correlated with clinical cure. This case contributes to the limited clinical evidence for managing infections caused by emerging CR *S.* Kentucky.

## Case presentation

A 44-year-old man was admitted on 19 June 2024 with a one-month history of cough and fatigue. Complete blood count showed trilineage cytopenia: leukopenia (WBC 2.4 × 10^9^ /L), anemia (HGB 77 g/L), and thrombocytopenia (PLT 75 × 10^9^ /L). Bone marrow examination and MICM (morphology, immunology, cytogenetics, and molecular biology) workup confirmed a diagnosis of acute erythroid leukemia (AML-M6b). Next-generation sequencing identified mutations in *NPM1* (nucleophosmin 1, 38.3%) and *PTPN11* (protein tyrosine phosphatase non-receptor type 11, 11.7%). The DCAG chemotherapy regimen (decitabine, cytarabine, aclarubicin, G-CSF) was initiated on 22 June 2024. Oral sitafloxacin was administered for antimicrobial prophylaxis.

The patient developed a cough on 27 June 2024, with elevated inflammatory markers: C-reactive protein (CRP) 11.29 mg/L (↑), procalcitonin (PCT) 0.075 ng/mL (↑), and serum amyloid A (SAA) 21.6 mg/L (↑). Subsequently, he developed fever (39 ℃) and productive cough on 28 June 2024. Sputum was collected when expectorated, and two sets of blood cultures were drawn at peak fever; both were submitted to the microbiology laboratory. Antibiotic therapy was empirically escalated from sitafloxacin to intravenous cefoperazone-sulbactam. However, the patient showed no clinical improvement. On 30 June 2024, due to worsening symptoms and rising inflammatory markers, antimicrobial coverage was broadened to meropenem and vancomycin, with isavuconazole added for antifungal prophylaxis. Blood and sputum cultures obtained during this period returned negative, yet inflammatory markers continued to rise and clinical symptoms persisted. Moreover, on 2 July 2024, the patient developed diarrhea and multiple rashes on the extremities. Consequently, respiratory pathogen testing and stool culture were simultaneously submitted. On the same day, the antibiotic regimen was further escalated to ceftazidime-avibactam, teicoplanin, and linezolid. Weakly positive *Mycoplasma pneumoniae* IgM antibody (±) prompted the addition of azithromycin to this combination. However, fever and cough persisted. Testing for common respiratory pathogens (influenza A and B viruses, parainfluenza virus, adenovirus, respiratory syncytial virus, bocavirus, coronavirus, rhinovirus, and *Chlamydia*) was negative. Blood tests showed: WBC 2.49 × 10^9^ /L (↓), NEU 0.57 × 10^9^ /L (↓), RBC 1.47 × 10^12^ /L (↓), HGB 45 g/L, and PLT 51 × 10^9^ /L (↓). Inflammatory markers continued to rise: CRP 80.27 mg/L (↑) and SAA 431.3 mg/L (↑). On 6 July 2024, the patient experienced aggravated cough and rash. Blood tests revealed: WBC 3.21 × 10^9^ /L (↓), NEU 0.22 × 10^9^ /L (↓), RBC 1.98 × 10^12^ /L (↓), HGB 59 g/L (↓), PLT 30 × 10^9^ /L (↓), consistent with chemotherapy-induced bone marrow suppression. Inflammatory markers progressively increased: SAA 352.3 mg/L (↑) and CRP 80.27 mg/L (↑). During this period, routine stool testing and *Clostridioides difficile* culture were performed; the latter returned negative results, effectively excluding antibiotic-associated diarrhea as the primary cause of his gastrointestinal symptoms.

The critical value report of stool culture was received from our laboratory on July 8, 2024: *S.* Kentucky (serotype I 8,20:i: z6) was identified by antigen-antibody agglutination testing using a commercial diagnostic serum kit. Antimicrobial susceptibility testing revealed a MDR phenotype, resistant (R) to aminopenicillins and third and fourth-generation cephalosporins (minimum inhibitory concentration [MIC] in mg/L: ampicillin > 16 [R], ampicillin/ sulbactam > 16/8 [R], piperacillin/tazobactam > 64/4 [R], ceftriaxone [R], cefotaxime > 32 [R], ceftazidime > 16 [R], cefepime > 16 [R]) and quinolones (ciprofloxacin > 2 [R], moxifloxacin > 4 [R]). Furthermore, this strain was resistant to carbapenems (imipenem > 8 [R], meropenem > 8 [R]), but susceptible to tigecycline ≤ 2 [susceptible, S], polymycin ≤ 0.5 [S], and ceftazidime-avibactam 21 mm (“Kirby-Bauer method” diameter, S). An immunochromatographic assay confirmed NDM production. Subsequent synergy testing demonstrated a potent effect for the combination of aztreonam and ceftazidime-avibactam. Based on these conclusive microbiological results, the antimicrobial regimen was rationally optimized on July 9 to a targeted combination of aztreonam and ceftazidime-avibactam, and other antibiotics were discontinued. Isavuconazole was continued because the fungal culture had yielded *Candida glabrata.*

**Fig. 1:**
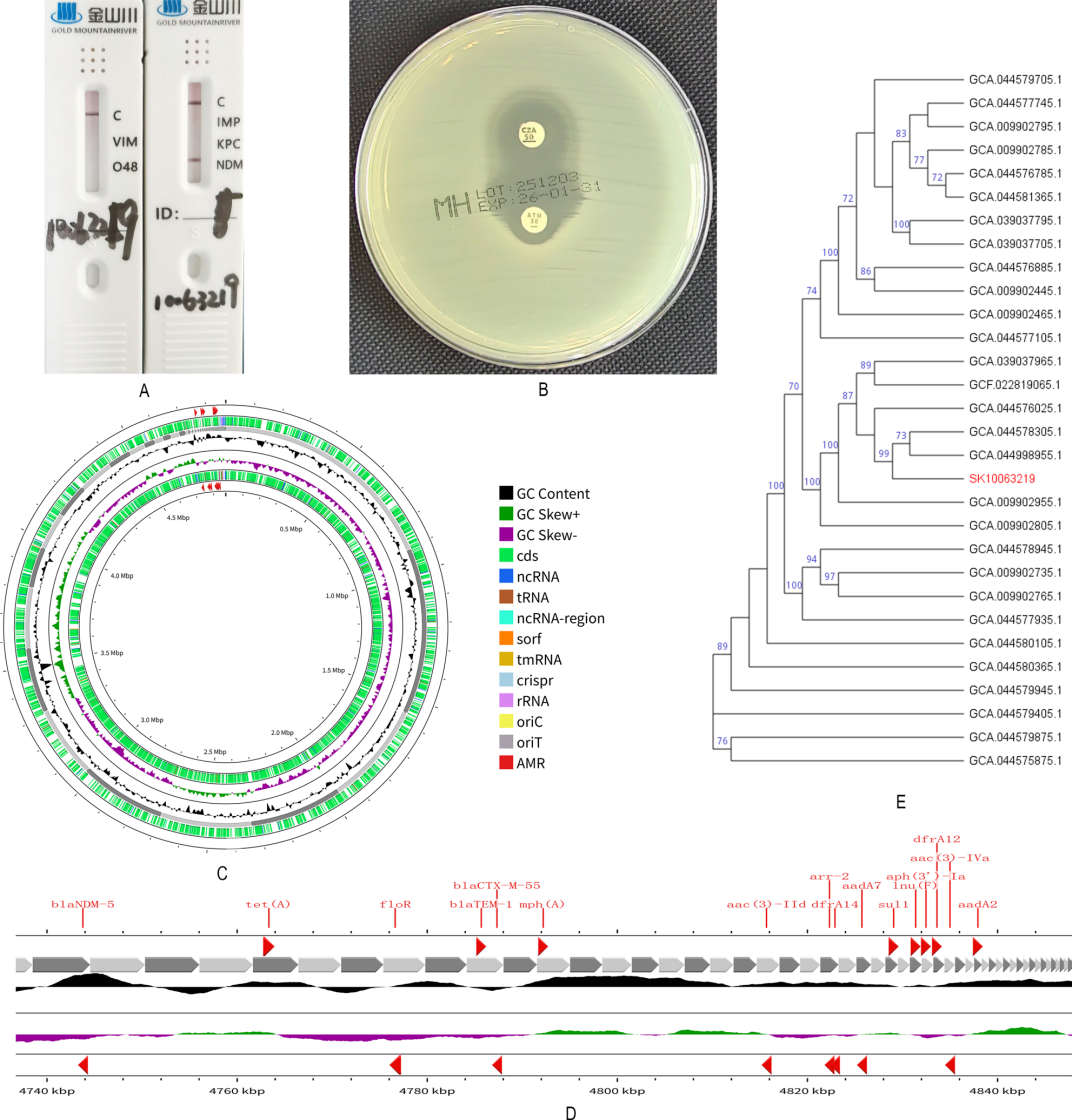
Characterization of the CR *S.* Kentucky isolate. **A**. Results of the immunochromatographic assay for carbapenemase detection. **B**. Results of the combination disk synergy test (aztreonam [ATM] plus ceftazidime-avibactam [CZA]). **C**. Circular genome map of the CR *S.* Kentucky strain SK10063219. **D**. Enlarged view of the genomic region (black box in panel **C**) harboring key antimicrobial resistance determinants. **E**. Phylogenetic tree of SK10063219 (highlighted in red) and other *S*. Kentucky strains isolated from China

Following the initiation of targeted therapy with aztreonam and ceftazidime-avibactam on July 9, the patient’s clinical condition improved markedly. Defervescence occurred by 12 July 2024, accompanied by the resolution of respiratory symptoms, rash, and diarrhea. Inflammatory markers (CRP, PCT, SAA) normalized progressively. Microbiological clearance was confirmed through repeated testing: although a stool culture on 13 July 2024 remained positive for *S.* Kentucky, a subsequent culture on 18 July 2024 subsequently yielded no *Salmonella* growth. Serial blood cultures also remained negative. Given the sustained clinical and microbiological response, the patient was discharged on 18 July 2024 with a plan for outpatient antibiotic completion. A detailed timeline of the clinical course is provided in Table 1. Enzyme identification and synergy test results are presented in Fig. 1A and B.

WGS of the isolate was performed on an Illumina NextSeq platform. Bioinformatic analysis using ABRicate aganist the ResFinder 4.1, CARD, NCBI and mob-suite databases identified the chromosomally located resistance genes blaNDM-5, blaCTX-M-55, and blaTEM-1. This genetic profile correlates with the observed resistance phenotype. Multilocus sequence typing (MLST) determined the strain as sequence type ST198. A genomic map and a summary of resistance determinants are provided in Fig. 1C and D. Further in-depth plasmid analysis was performed using MOB-suite version 3.1.9. The genome assembly of this strain consists of 53 scaffolds. Among these, four scaffolds carry complete plasmid backbones (see Table S1). All four plasmid elements are integrated into the chromosome rather than existing as free plasmids. These plasmid regions all contain replicons, relaxase genes, and oriT sites, indicating their potential mobilization and horizontal transfer ability. A phylogenetic tree including SK10063219 (this study) and other *S. Kentucky* strains from China (see Table S2 for strain information) was constructed by the neighbor-joining method using TreeBeST. The tree revealed that SK10063219 was closely related to strains GCA_044578305.1 and GCA_044998955.1, both of which were ST198 *S. Kentucky* strains isolated from chickens in China (Fig. 1E).

**Table 1:** Timeline of inflammatory markers, microbiology tests and treatment

Date	WBC (/L)	PCT (ng/mL)	CRP (mg/L)	SAA (mg/L)	Microbiology test	Antimicrobial treatment
6.19	6.33 × 10^9	0.068(↑)	6.5(↑)	/	/	6.22 STX
6.25	6.49 × 10^9	0.088(↑)	5.47	5.5	/	/
6.27	3.25 × 10^9(↓)	0.075(↑)	11.29(↑)	21.6(↑)	6.28–7.4 Sputum culture (-) Blood culture^#^ (-)	/
6.29	2.47 × 10^9(↓)	0.111(↑)	63.09(↑)	239.6(↑)	/	6.28 SCF
6.30	2.85 × 10^9(↓)	0.113(↑)	62.11(↑)	269.7(↑)	/	MEM + VA+ISA
7.1	3.1 × 10^9(↓)	0.116(↑)	72.39(↑)	319.5(↑)	/	/
7.2	2.62 × 10^9(↓) NEU 0.42 × 10^9(↓)	0.072(↑)	85.61(↑)	367.7(↑)	Common respiratory pathogenic bacteria*(-) MP IgM(±) G test(-)	CZA + TEC+LZD + AZM
7.3	2.49 × 10^9(↓)	0.048	80.27(↑)	431.3(↑)	/	/
7.5	3.21 × 10^9(↓)	0.036	66.04(↑)	352.3(↑)	7.5–7.9 Feces culture: CRSK+*Candida parapsilosis*	/
7.7	2.65 × 10^9(↓)	0.054(↑)	54.58(↑)	525(↑)	feces culture: *Clostridioides difficile* (-)	/
7.9	1.58 × 10^9(↓)	0.06(↑)	112.21(↑)	566.1(↑)	G test(-) MP IgM(±)	7.9 CZA + ATM+ISA
7.10	1.88 × 10^9(↓)	0.06(↑)	85.47(↑)	482.7(↑)	7.10–7.13 Feces culture: CRSK+*candida glabrata* 7.10–7.15 Blood culture (-)	/
7.12	2.20 × 10^9(↓)	0.044	26.8(↑)	102.6(↑)	/	/
7.14	2.51 × 10^9(↓)	0.04	8.01(↑)	18.4(↑)	/	/
7.16	3.43 × 10^9(↓)	0.044	3.9	6.5	/	/
7.18	4.2 × 10^9	0.036	3	/	Feces culture: *salmonella* (-)	/

Note: An upward arrow (↑) indicates a positive result or elevated level of the tested parameter, whereas a downward arrow (↓) signifies a decreased result. “-” represents a negative result; # Blood culture was performed using two aerobic and two anaerobic blood culture bottles; * Influenza A and B virus, parainfluenza virus, adenovirus, respiratory syncytial virus, bocavirus, coronavirus, rhinovirus, chlamydia

Abbreviations: CRSK: carbapenem-resistant *S*. Kentucky; STX: Sitafloxacin; SCF: Cefoperazone-sulbactam; MEM: meropenem; VA: vancomycin; ISA: Isavuconazole; CZA: ceftazidime-avibactam; TEC: teicoplanin; LZD: linezolid; AZM: azithromycin; ATM: aztreonam

## Discussion and conclusions

AML-M6, particularly the M6b subtype, is an aggressive and uncommon form of acute myeloid leukemia, accounting for less than 1% of all AML cases and carrying a very poor prognosis, with a median survival of approximately three months [7]. In patients with hematological malignancies, chemotherapy-induced immunosuppression increases the risk of infections, which can contribute to treatment failure and mortality. Among these infections, *Salmonella* stands out as a virulent pathogen, and the emergence of CR strains further escalates the therapeutic challenge. Notably, previous reports indicate that the incidence of *Salmonella enterica* increases in febrile AML patients following chemotherapy [7]. Hence, the successful management of CR *Salmonella* infections in this high-risk population is critical to improving patient outcomes.

Two recent observations are relevant to our patient. First, a multicenter study of patients with advanced cancer and COVID-19 pneumonia showed that mortality was driven by profound immunosuppression rather than viral load [8]. This reinforces the principle that intensive, combination regimens guided by real-time susceptibility testing are required when highly resistant organisms infect immunocompromised hosts. Second, SARS-CoV-2 infection or vaccination can trigger persistent immune dysregulation, gut barrier disruption and transient enrichment of CRE (reviewed in [9]). Although the patient had received three doses of COVID-19 vaccine, we lacked information regarding whether he experienced long COVID. Furthermore, he tested negative for SARS-CoV-2 by PCR during the present admission, therefore we couldn’t assess any influence of COVID-19 vaccination or viral evolution on his clinical course.

*Salmonella*, a member of the Enterobacteriaceae family, is a major food-borne pathogen typically transmitted to humans through the consumption of contaminated poultry, eggs, and water. While over 2,600 serotypes have been identified, *S.* Enteritidis and *S*. Typhimurium remain the most frequent causes of human infection [10]. *S.* Kentucky is also a common cause of NTS. The clinical manifestation of salmonellosis depends critically on the host’s immune status. In immunocompetent individuals, infection is often self-limiting, managed with supportive care like fluid and electrolyte replacement, whereas immunocompromised patients invariably require antimicrobial therapy. The first-line therapeutic agents for invasive *Salmonella* infections have traditionally included fluoroquinolones (e.g., ciprofloxacin), third-generation cephalosporins, and azithromycin. The WHO 2024 priority pathogen list underscores the significant threat of antimicrobial resistance, ranking CRE as a critical priority and fluoroquinolone-resistant NTS as a high priority [11].

CR *Salmonella enterica* strains producing various enzymes, including KPC, NDM, IMP, VIM, and OXA-48, have been previously documented [12–16]. Rapid identification of the specific enzyme is therefore critical for guiding targeted therapy. Immunochromatographic assays provide a swift solution, capable of simultaneously detecting common carbapenemases (KPC, NDM, IMP, VIM, OXA-48) within 20 min with sensitivity and specificity exceeding 90% [17]. This allows for the early initiation of precise antimicrobial regimens. NDM-5 is a subtype of NDM. Among these, the NDM-5 variant is most frequently detected in *Escherichia coli* and *Klebsiella pneumoniae*, with only sporadic reports in *Salmonella enterica* [18, 19], primarily in the *S.* Typhimurium serovar [10, 18]. The NDM-5-producing *S*. Kentucky strain isolated from our patient represents a notable addition to these unusual cases.

Ceftazidime-avibactam is a key therapeutic agent in this context. Ceftazidime-Avibactam inhibits class A (including KPC), class C, and some class D (e.g., OXA-48-like) β-lactamases but is inactive against class B metallo-β-lactamases (MBLs). Aztreonam, a monobactam, remains stable against MBLs. In vitro studies have demonstrated a synergistic effect between aztreonam and ceftazidime-avibactam against MBL-producing strains. Corresponding clinical evidence suggests that combination therapy with these two agents significantly reduces mortality in patients with MBL-CRE bloodstream infections compared to other regimens [20, 21]. In this case, a patient with leukemia developed a complex intestinal *Salmonella* infection following chemotherapy. Rapid enzymatic testing confirmed the production of an MBL. Subsequent WGS identified the specific enzymes as NDM-5, CTX-M-55, and TEM-1, which corresponded with the observed resistance phenotype. Guided by these findings, a combination regimen of aztreonam and ceftazidime-avibactam was successfully implemented, resulting in improved infection control. This underscores the critical role of integrating rapid enzyme identification and synergy testing to guide effective, mechanism-based therapy for infections caused by such challenging MDR pathogens.

A particularly intriguing aspect of this isolate concerns its fluoroquinolone resistance. While common plasmid-mediated determinants (such as *qnr*genes, *aac(6’)-Ib-cr*, and *qepA* [21].) were absent, re-analysis using the NCBI Pathogen Detection platform and AMRFinderPlus confirmed the presence of chromosomal point mutations. Specifically, we identified *gyrA* (S83F and D87N)​ and *parC* (S80I), which are canonical mutations conferring fluoroquinolone resistance in *Salmonella*.

Interestingly, beyond these target-site mutations, our genomic analysis also revealed the presence of the *sdiA* gene (99.49% identity), a quorum-sensing regulator known to upregulate the AcrAB efflux system [22]. While the primary mechanism of resistance in this strain is unequivocally linked to the *gyrA* and *parC* mutations, the potential contribution of *sdiA*-mediated efflux to the overall resistance profile warrants further investigation. This case highlights the necessity of combining PMQR screening with point mutation analysis for accurate genotypic prediction of fluoroquinolone resistance.

This study has several limitations inherent to its retrospective, single-case design. First, the lack of data on the immune impact of the COVID-19 pandemic on patients and gut microbiota composition precluded assessment of whether pandemic-related immune dysregulation or an altered intestinal microbiome predisposed him to colonization by CRE, which may have been the source of the blaNDM-5-positive Salmonella isolate. Similarly, it was not possible to determine whether this infection resulted from the reactivation of a latent strain or was acquired nosocomially. Second, a new antibiotic regimen should be initiated on the basis of robust microbiological evidence; incomplete data compromise treatment success. Third, Finally, unlike the typical plasmid-borne blaNDM-5, our strain carried this gene chromosomally; its transmissibility across species and underlying mechanisms warrant further investigation.

In conclusion, we report an unusual case of CR *S.* Kentucky harboring blaNDM-5, blaCTX-M-55, and blaTEM-1, a resistance gene profile previously observed in *S.* Typhimurium but exceptionally uncommon in this serovar [14]. This case underscores the critical role of combining molecular diagnostics, which elucidate the resistance mechanism, with synergistic antimicrobial susceptibility testing to guide effective therapy, especially in the absence of formal guidelines for CR *Salmonella*. The treatment protocol optimized by our team, informed by this approach, successfully cleared the infection and offers a valuable management strategy for high-risk AML patients facing similar therapeutic challenges.

## Supplementary Information

Below is the link to the electronic supplementary material.

Supplementary Material 1

Supplementary Material 2

Supplementary Material 3

Supplementary Material 4

Supplementary Material 5

Supplementary Material 6

## Data Availability

All the data supporting this case report have been included in the manuscript text. The whole-genome sequencing results have been deposited in National Center for Biotechnology Information (accession number: JBOEPZ000000000) that are publicly accessible at (https:/www.ncbi.nlm.nih.gov/nuccore/JBOEPZ000000000.) The relevant Bioproject number and Biosample number are PRJNA1265139 and SAMN48586150, respectively. The raw sequencing data for this sample have been deposited in the Sequence Read Archive (SRA) under accession number SRR37140338.

## Abbreviations

*S*. Kentucky: *Salmonella* enterica serotype Kentucky
MDR: Multi-drug resistance
AML-M6: Acute erythroid leukemia
CR: Carbapenem-resistant
WGS: Whole genome sequencing
NTS: Non-typhoidal *Salmonella*
CRE: Carbapenem-resistant *Enterobacteriaceae*
ESBLs: Extended-spectrum β-lactamases
CRP: C-reactive protein
PCT: Procalcitonin
SAA: Amyloid A
NEU: Neutrophils
MBLs: Class B metallo-β-lactamases
