# Supplementary material for: Successful treatment of an NDM-5-producing, carbapenem-resistant Salmonella Kentucky ST198 infection with aztreonam and ceftazidime-avibactam in a patient with acute erythroid leukemia: a case report
Source: BMC Infect Dis. 2026 Jun 10;26:1224. doi: 10.1186/s12879-026-13519-9 (PMC13330401; doi:10.1186/s12879-026-13519-9)
Supplement: Supplementary file 6 — Supplementary Material 6 [file 12879_2026_13519_MOESM6_ESM.docx]

**Table S1 Characteristics of identified plasmid scaffolds**

| Contig | Length | Replicon | Relaxase (Mob) | oriT | Mobility |
| --- | --- | --- | --- | --- | --- |
| Scaffold21 | 18415 | IncQ1 | MOBQ | Yes | Mobilizable |
| Scaffold35 | 5063 | rep2350 | MOBP | Yes | Mobilizable |
| Scaffold36 | 4927 | ColRNAI | MOBP | Yes | Mobilizable |
| Scaffold37 | 4361 | Col156 | MOBQ | Yes | Mobilizable |

**Table S2 Genome sequence information of strains used for phylogenetic analysis**

| **strain** | **GenBank assembly accession** | **MLST** | **Year** | **geographic origin** | **Source** |
| --- | --- | --- | --- | --- | --- |
| sc-h42 | GCA_009902765.1 | 198 | 2013 | Sichuan | Human |
| sc-h43 | GCA_009902735.1 | 198 | 2013 | Sichuan | Human |
| zj-h16 | GCA_009902465.1 | 198 | 2016 | Zhejiang | Human |
| hn-h2 | GCA_009902785.1 | 198 | 2017 | Hunan | Human |
| hn-h4 | GCA_009902795.1 | 198 | 2016 | Hunan | Human |
| zj-h17 | GCA_009902445.1 | 198 | 2017 | Zhejiang | Human |
| gx-h1 | GCA_009902805.1 | 198 | 2015 | Guangxi | Human |
| fj-h1 | GCA_009902955.1 | 198 | 2017 | Fujian | Human |
| s202011-132 | GCA_039037705.1 | 198 | 2020 | Guangzhou | Chicken |
| s202011-126 | GCA_039037795.1 | 198 | 2020 | Guangzhou | Chicken |
| s202011-118 | GCA_039037965.1 | 198 | 2020 | Guangzhou | Chicken |
| 29 | GCA_044575875.1 | 314 | 2018 | China | Chicken |
| 21 | GCA_044576025.1 | 198 | 2017 | China | Human |
| 59 | GCA_044576785.1 | 198 | 2018 | China | Human |
| 53 | GCA_044576885.1 | 198 | 2018 | China | Human |
| 55 | GCA_044577105.1 | 198 | 2018 | China | Human |
| 202103-QX-8 | GCA_044577745.1 | 198 | 2021 | China | Chicken |
| H204 | GCA_044577935.1 | 198 | 2015 | China | Human |
| s202011-18 | GCA_044578305.1 | 198 | 2020 | China | Chicken |
| 11-SH | GCA_044578945.1 | 198 | 2010 | China | Chicken |
| 15091304 | GCA_044579405.1 | 314 | 2015 | China | Chicken |
| 05-6 | GCA_044579705.1 | 198 | 2020 | China | Chicken |
| 59-SH | GCA_044579875.1 | 314 | 2013 | China | Human |
| 77-SH | GCA_044579945.1 | 314 | 2016 | China | Chicken |
| H200 | GCA_044580105.1 | 314 | 2016 | China | Human |
| 73 | GCA_044580365.1 | 314 | 2018 | China | Chicken |
| 115 | GCA_044581365.1 | 198 | 2019 | China | Human |
| s202011-11-complete | GCA_044998955.1 | 198 | 2020 | China | Chicken |
| NT-h3190 | GCF_022819065.1 | 198 | 2021 | Jiangsu | Human |
| SK10063219 | this study | 198 | 2024 | Guangzhou | Human |
